# Numerical characterization of astronaut CaOx renal stone incidence rates to quantify in-flight and post-flight relative risk

**DOI:** 10.1038/s41526-021-00187-z

**Published:** 2022-01-28

**Authors:** Debra A. Goodenow-Messman, Suleyman A. Gokoglu, Mohammad Kassemi, Jerry G. Myers

**Affiliations:** 1 National Aeronautics and Space Administration - John H. Glenn Research Center, Cleveland, OH USA; 2 National Center for Space Exploration Research (NCSER), Cleveland, OH USA

**Keywords:** Biomarkers, Risk factors

## Abstract

Changes in urine chemistry potentially alter the risk of renal stone formation in astronauts. Quantifying spaceflight renal stone incidence risk compared to pre-flight levels remains a significant challenge for assessing the appropriate vehicle, mission, and countermeasure design. A computational biochemistry model representing CaOx crystal precipitation, growth, and agglomeration is combined with a probabilistic analysis to predict the in- and post-flight CaOx renal stone incidence risk ratio (IRR) relative to pre-flight values using 1517 astronaut 24-h urine chemistries. Our simulations predict that in-flight fluid intake alone would need to increase from current prescriptions of 2.0–2.5 L/day to ~3.2 L/day to approach the CaOx IRR of the pre-flight population. Bone protective interventions would reduce CaOx risk to pre-flight levels if Ca excretion alone is reduced to <150 mg/day or if current levels are diminished to 190 mg/day in combination with increasing fluid intake to 2.5–2.7 L/day. This analysis provides a quantitative risk assessment that can influence the critical balance between engineering and astronaut health requirements.

## Introduction

Spaceflight, specifically the exposure to microgravity and the situational conditions imposed by launch, living in space, and return to a terrestrial gravitational environment, induce numerous alterations in astronaut physiology^1^. As described in the NASA Human Research Roadmap^2^, physiological changes alter the risk to astronaut health and performance requiring countermeasures, i.e., treatments and other measures employed to counter one or more detrimental physiological or psychological effects of spaceflight’s altered environmental conditions^3,4^, to mitigate safety concerns^5–7^. These risks include the potential for in-flight symptomatic renal stones, where limited treatment may jeopardize the astronauts’ health and could endanger the space mission. In the 1980s, a cosmonaut onboard the Mir spacecraft described detailed symptoms and reduction in the ability to perform operations that has since been attributed to the formation and spontaneous resolution (passage) of a renal ureteral stone^8^. US astronauts do not have an immunity to this risk, although no in-flight stone incidence has yet occurred on U.S. space vehicles. Pietrzyk^9^ reports that there have been 14 symptomatic renal stone events in 5434.5 person-years as of 2008; 7 pre-flight, 7 post-flight, and 0 in-flight. Of stones collected, calcium oxalate (CaOx) made up approximately 26%, uric acid 7%, mixed components 7%, and unknown constituents 60%^1^. Notably, an astronaut’s post-flight prevalence of symptomatic renal stone exceeds that of the general US non-stone-forming population^9^.

**Fig. 1:**
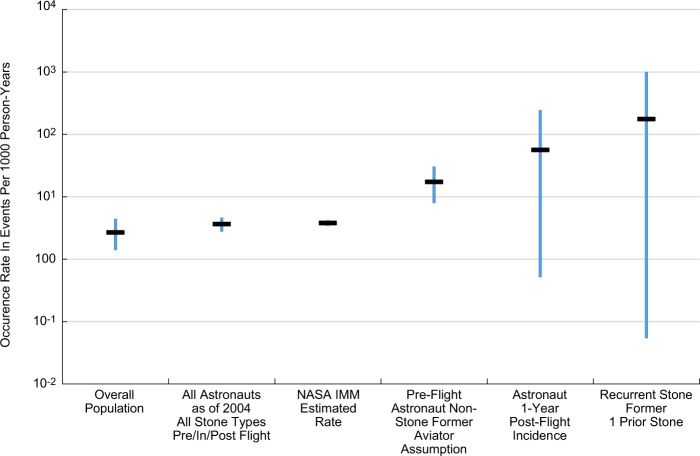
Comparison of estimated symptomatic calcium kidney stone occurrence rates for astronaut risk. Comparing occurrence rates, including first presentation incidence rates, evaluated for the overall population^13^, all pre-flight, in-flight, post-flight^9^ astronauts, the NASA IMM Risk Model^1^, analogous pre-flight astronaut non-stone former aviator assumption^52^, and 1-year post-flight astronauts to recurrent stone formers^16^ illustrates the degree of ambiguity possible in predicting astronaut symptomatic calcium kidney stone formation rates. The calculations for the 1-year post-flight astronaut rate are described in the “Methods” and tabulated in Table 4 with all population incidence and recurrence rates identified for this study. The overall population rate is below the astronauts’ rate. This is to be expected, considering the astronaut population has a higher proportion of males than the general population^91^, and males have a higher incidence rate of renal stones^13^. Additionally, the various astronaut incidence rate estimates either include post-flight astronauts or are premised on analogous aviator population data that have a higher incidence of renal stones^52^, as shown in the “Methods”.

Pre-acceptance screening of medical histories is the key to ensure that individuals selected into the astronaut corps belong to the non-stone former clinical category^10,11^. After acceptance, regular review of urinary system risk factors^12^ and observed symptomatic stone occurrences also place flight-ready astronauts into the non-stone former clinical category in the 5-year period preceding flight. Figure 1 illustrates how published observations of CaOx stone incidence rates compare for important flight-status milestones in an astronaut’s career.

As illustrated in Fig. 1, observed and analogs pre-flight incidence rates, exceed the general population rates estimated in the Rochester epidemiological study^13,14^. The “zero” current observations of in-flight symptomatic stones in US astronauts infer little change in the predicted incidence rate when premised on pre-flight incidence rate priors^1^. However, as shown later, combining observations from the 1-year post-flight symptomatic stones^1^ with observations of clinical risk stemming from changes in post-flight urine supersaturation^9,15^ suggests that 1-year post-flight astronauts experience incidence rates of 2–7 times that of pre-flight estimates. In comparison, estimates of the terrestrial population single and multiple recurrent stone former occurrence rates have the potential to be 10 and 45 times the astronaut pre-flight estimated incidence rates, respectively^16^. This implies that astronauts likely experience an increase in the in-flight risk of stone formation, but not to the level clinically seen in terrestrial recurrent stone formers.

The time and exact metabolic process for an individual developing a calcium stone is not well understood and depends, among other things, on the interaction of calculus with renal tissue (plaques and tubule plugs) and on the role of calcium salt supersaturation, precipitation and crystal interactions^17,18^. Due to skeletal unloading and space operational limitations, in-flight and post-flight astronauts exhibit higher urinary calcium (mg/day) and lower urine volume output compared to pre-flight astronauts. Oxalate and citrate may also be altered depending on in-flight dietary factors^9,19,20^. In-flight studies identify an increase in urinary CaOx supersaturation as an increase in the risk of an in-flight symptomatic renal stone occurrence. Urine chemistry studies of Space Shuttle astronauts’^9,21–23^, show that 25% of astronauts exhibit elevated CaOx supersaturation pre-flight compared to 46% of astronauts post-flight, with male astronauts and male analog cohorts exhibiting more susceptibility to elevated urine CaOx supersaturation than their female counterparts^19,24^. Hydration, exercise, and nutritional countermeasures represent the primary means to prevent elevated urine calcium supersaturation. Increased fluid intake, leading to increased urine volume, represents a potentially effective countermeasure to astronaut renal stone risk^25–27^. However, operational limits related to spaceflight resource mass, volume, and operational time required to maintain intake represent a significant challenge to this approach for in-flight astronauts^20,21^. High loading resistive exercise to mitigate calcium excretion from bone deconditioning by increasing osteocyte-derived negative reabsorption appears to have only a marginal effect as a renal stone occurrence countermeasure^20,28^. Flight astronaut and ground analog population studies indicate that potassium citrate therapy may represent an effective countermeasure as such therapy modulates elevated CaOx and other stone-forming precipitants’ supersaturation in over 10% of the tested populations^29,30^. Pharmacological interventions with antiresorptive bisphosphonates to protect bone health^31^ also show promise in mitigating excessive urine Ca excretion in astronauts, potentially by an average reduction between 30 and 125 mg/day as seen in 6-months spaceflight studies^32^.

For spaceflight missions, the reliance of risk characterization of renal stone formation by measures of urine supersaturation of calcium stone-forming salts^33^ generally follows the clinical guidelines^34^ as this qualitatively captures integrated effects on stone formation risk^35^. A recent set of studies by Kassemi and Thompson^36,37^ proposed an approach that potentially enhances the predictive and integrative capabilities of the urine supersaturation risk characterization. Typical urine supersaturation measures utilize computational systems, like EQUIL2^38^ and JESS^39^, that achieve chemical speciation via assessments of chemical and thermodynamic equilibrium calculations. Although the relative supersaturation scales may differ, these computational systems have recently been shown to predict the relative reduction in risk due to dietary impacts to citrate, potassium, and magnesium^40^. The Kassemi and Thompson^36,37^ approach utilizes a Population Balance Equation (PBE) based computational simulation model to augment chemical speciation. This approach captures the physics behind precipitation, nucleation, species transport, crystal growth kinetics in a fluid stream, and the agglomeration/breaking interactions between single species CaOx crystals. The simulation estimates the changes in the population of stone sizes, with effective diameters on the order of microns (1.0E−06 m) to mm (1.0E−03 m), due to spaceflight-induced variations in urine chemistry by considering these factors. Analysis with this technique utilizing characteristic urine chemistries of terrestrial and spaceflight non-stone formers (NSF) and stone formers (SF) elucidated a nonlinear relation between renal stone calcium and oxalate constituents, where apparent risk, noted as the size of the largest single stone in 1 mL of free fluid, could increase several times for relatively small deviations from normal urine chemistry^36^. Similarly, evaluation of dietary countermeasures, such as increasing citrate and urinary output levels, induces effective inhibition of large stone formation^37^.

Even with the evidence of negatively altered urine supersaturation of stone-forming salts during spaceflight and the observed post-flight occurrences in US astronauts, the question “What renal stone risk do astronauts experience during spaceflight and how much can interventions mitigate that risk?” needs to be addressed to inform spaceflight risk in a manner congruent with engineering analysis^41^. In this study, we address the question of predicting astronaut renal first-stone incidence rates by implementing the Kassemi and Thompson^36,37^ PBE model. The PBE model explicitly considers two major factors that drive stone nucleation and growth from both thermodynamic and kinetics perspectives: urine chemistry free-energy driving precipitation from supersaturation of dissolved salts; and kinetic (rate-limiting) processes associated with the growing crystal^18,42^. This is integrated into a probabilistic framework and trained with individualized urine chemistries known from NSF, SF, pre- and post-flight astronauts. From this integrated system, we present comparisons to terrestrial studies of stone-forming populations, to illustrate the system’s fidelity, and predictions of astronaut renal stone incidence rate ratios, to illustrate the integrated framework’s utility in addressing the relative impact of spaceflight risk factors.

## Results

### Modeling process characterization and validation

**Fig. 2:**
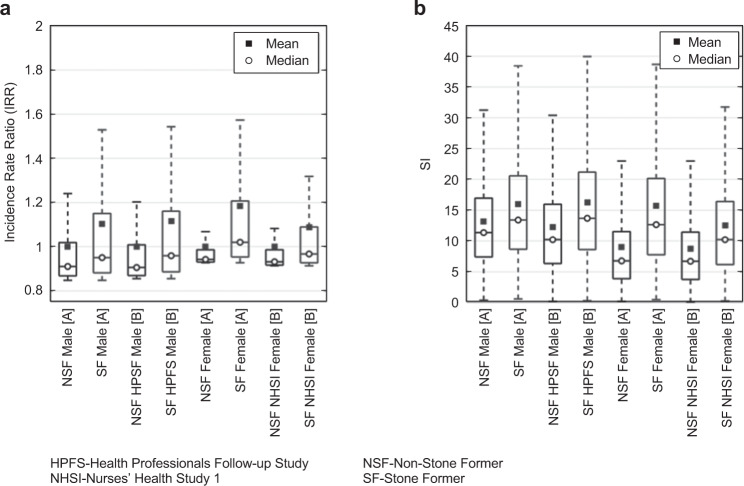
Characterization of numerical predictions for known stone formers (SF) and non-stone formers (NSF) population urine chemistries. The model’s ability to predict differences in SF and NSF populations is illustrated by contrasting male and female stone formers (SF) and non-stone formers (NSF) population urine chemistries from case and control sources [A]^60^ and [B]^45^. In **a**, each numerically predicted IRR distribution is normalized using the case and control pair’s control group mean predicted IR as the reference. In **b**, the SI of each case and control pair, as determined by JESS, is presented. SI values in **b** are not normalized by the control mean of each pair.

Figure 2 illustrates our modeling analysis in characterizing predicted incidence risk ratio (IRR) and JESS saturation index (SI)^43,44^ of published terrestrial SF (case) and NSF (control) population urine chemistries. The IRR is defined as the ratio of predicted incidence rate to a reference incidence rate. In Fig. 2a, where each case–control pair is normalized to each control’s mean predicted incidence rate, the SF case mean IRR is 8 to 18% higher than that of NSF controls. The median is lower than the mean for both cases (−14 to −11%) and controls (−10 to −6%), resulting in the case median elevated above the control by 4–8%. The change in mean and median values is accompanied by a reduction in the skewness of the case distributions (skewness 1.9–2.8) from that of the controls (skewness 3.0–5.0), indicating more symmetric case relative risk distributions. Noticeably, the control population simulations do not extend above a maximum upper adjacent value (UAV) of IRR = 1.24 for males and 1.1 for females as indicated by the upper tail limit of each control case box plot. In contrast, the case population maximum UAV IRR always exceeds 1.3 and can reach as high as 1.57. As indicated in Fig. 2b, SI between controls and cases show similar trends with those of the IRR distributions, with mean and median case values elevated above those in each corresponding case. Relatively, in the SI values, the mean exceeds the median and the relative maximum values exceed 3 (control) and 4 (case) times each distribution mean. Notably, the control populations UAV do not exceed SI = 32 for males and 23 for females.

**Fig. 3:**
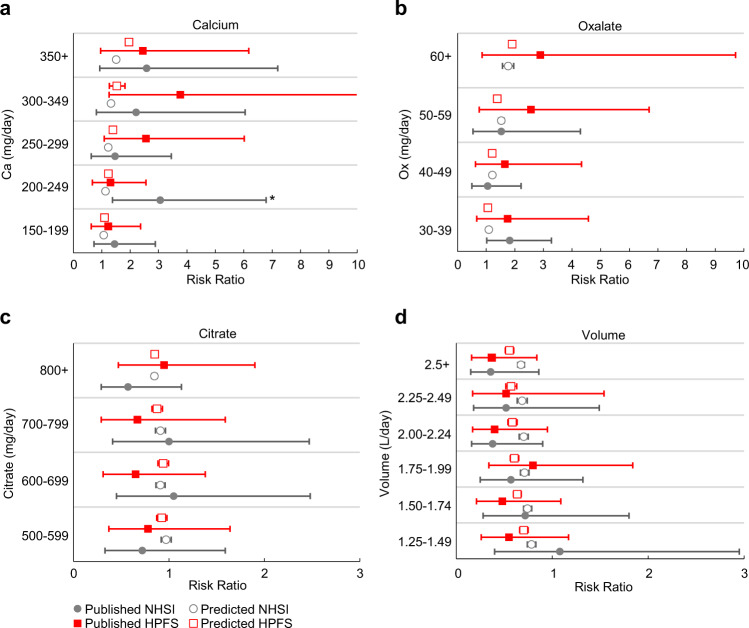
Comparisons of the published Curhan^45^ HPFS and NHSI populations relative risk ratios to numerically predicted risk ratios. The numerical estimates utilize sample populations derived from the published mean and standard deviation for each urine constituent: **a** calcium, **b** oxalate, **c** citrate, and **d** urine volume. The combined case’s and control’s urine constituent statistics were used to create a gamma distribution which was then sampled, to create 10,000 representative urine samples for the sample population predictions. Markers illustrate the mean value of each referent and predicted population and the whiskers represent the 95% confidence intervals of the mean. * indicates that the published and predicted pair show a statistically significant difference with *P* < 0.05.

To characterize the model’s fidelity in distinguishing change in relative risk, we reproduced the urine chemistry constituent case-to-control risk ratio (RRs) analysis published in Curhan^45^. We utilized the total population case and control data of the Nurses’ Health Study I (NHSI) and the Health Professionals Follow-up Study (HPFS) data sets from Curhan^45^ to produce 38 predicted incidence rate distributions and case-to-control risk ratios, as described in the methods sections. Figure 3 shows the risk ratios of the validation data set using the model analysis compared to the published NHSI and HPFS mean risk ratio’s 95th confidence interval (CI) associated with various excretion levels of calcium, oxalate, volume, and citrate. Utilizing the approach of Altman and Bland^46^ to determine the difference in two RR estimates, we can state that there is no strong evidence that the predictive and referent distributions are different in 37 of the 38 comparative pairs (i.e., *P* > 0.05). The calcium interval (Fig. 3a, NHSI, Ca = 200–249 mg/day) with *P* < 0.05, is below clinical elevated risk level of 250 mg/day. Given the relatively narrow range of the referent data, the RR observations and predictions match reasonably well, showing similar trends of increasing mean with increasing calcium or oxalate, as well as relative stability, with a mean RR close to 1, for both referent and predicted citrate and volume results.

### Estimation of astronaut IRR

We utilized the same process for characterizing the model analysis with referent sources to assess the renal stone risk to astronauts using the characteristic astronaut urine chemistry population data presented in Table 1. We utilized the model predicted pre-flight population mean IR (0.0085 per person-year) as the characteristic IR for all flight stage IRR calculations. As described in the methods, the calculated IRR value of the astronaut population analysis cannot exceed 2.43 as a result of preventing extrapolation outside the range of the regression curve.

**Table 1:** Pre-flight and post-flight urine concentrations used to train the model.

	Pre-flight urine		Post-flight urine	
	Mean (mg/day)	Standard deviation (mg/day)	Mean (mg/day)	Standard deviation (mg/day)
Concentrations				
Calcium	186.6	95.8	225.4	113.2
Oxalate	36.3	13.1	35.5	14.1
Citrate	711.2	379.0	627.2	329.5
Magnesium	115.2	74.1	100.8	76.6
Uric acid	640.4	219.7	567.6	243.5
Sulfate	2155.1	763.5	2306.5	872.3
Phosphate	1023.8	415.5	851.0	331.6
Sodium	7761.7	5860.7	6274.6	5544.6
Potassium	5154.4	3734.8	4234.5	3083.1
Additional urine characteristics				
Volume (L)	2.1	1.0	2.1	1.0
pH	6.1	0.4	5.8	0.5
Total no. of samples	508		433	

**Fig. 4:**
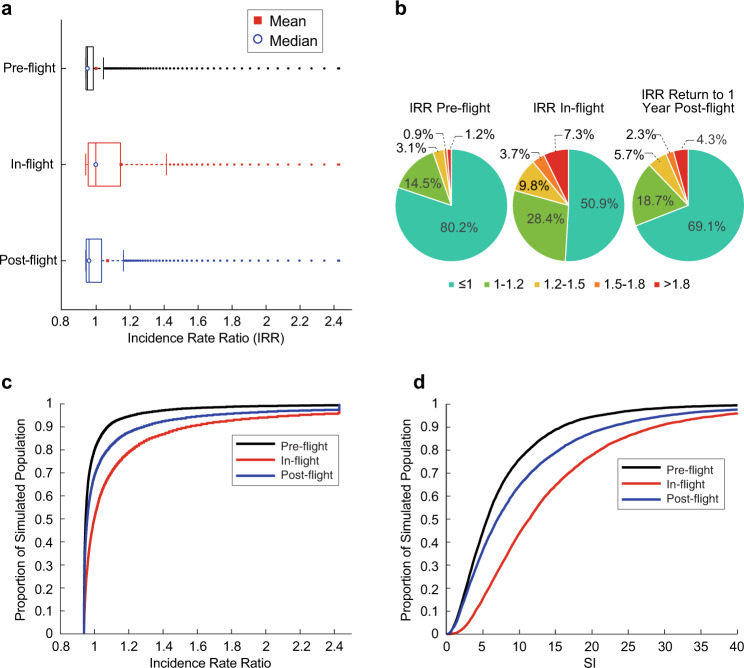
IRR distribution of the modeled astronaut population per flight phase. The predicted variation in renal stone risk for each simulated flight phase is shown following the renal stone risk analysis process described in the “Methods” section with **a** IRR distributions represented as box plots, **b** pie charts of the percentage of the simulated astronaut populations at select IRR intervals **c** cumulative density graphs of IRR, and **d** cumulative density graphs of SI for the simulated astronaut population. The estimated IR data is normalized to IRR using the predicted mean pre-flight incidence rate. We chose the IRR ranges in **c** to correspond to relatively important IRR ranges identified in the referent analysis or where natural cutoffs existed in the data set.

Illustrated in Fig. 4a, the analysis predicted IRR for pre-flight (1.00 ± 0.17 SD), in-flight (1.15 ± 0.35 SD: *P* < 0.001), and post-flight (1.07 ± 0.29 SD: *P* < 0.001) stages, with in-flight and post-flight distributions exhibiting higher mean, median and UAV values relative to pre-flight in a manner consistent with control and case studies of Fig. 2. As a means of representing the simulation outputs, Fig. 4b, c shows each flight phase’s results as binned pie charts showing discrete IRR intervals, and cumulative density plots respectively. The pre-flight astronaut population results predict 94.7% of the population with IRR values ≤ 1.2, which is similar to the terrestrial control population predictions shown in Fig. 2. In-flight, the predictions show that changes in urine chemistry result in 20.8% of the population with IRR > 1.2. Post-flight, the risk declines from in-flight to 12.3% of the total population with IRR > 1.2. In the 1 < IRR < 1.2 range, in-flight population increases by 13.9% from pre-flight and remains elevated by 4.2% post-flight. Cumulative density plots of SI in Fig. 4d illustrate that 95% of the pre-flight simulated population exhibits SI at or below 21. The in-flight and post-flight values at or below this SI = 21 level represent 80% and 89% of the populations, respectively.

### Relevance to clinical thresholds

**Fig. 5:**
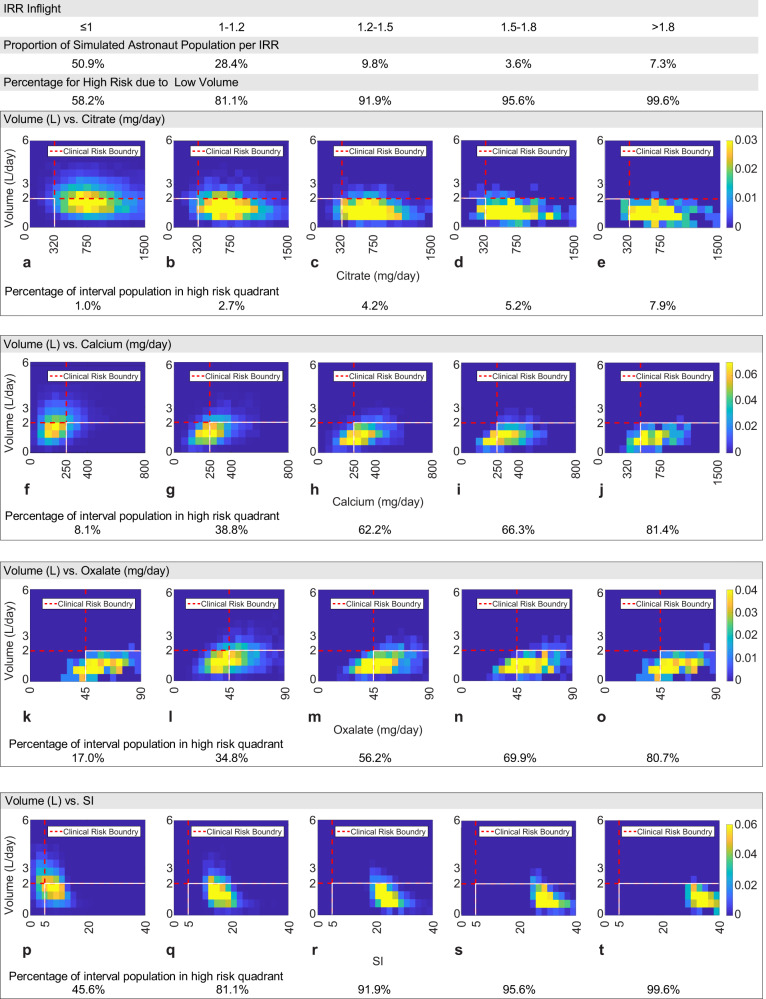
In-flight urine constituent concentration and CaOx Supersaturation Index (SI) heat maps at select IRR risk intervals. Each row of heat maps identifies the distribution of paired urine chemistry constituent data, while each column represents the percentage of the total simulated population that falls into that IRR interval. Calcium (**f**–**j**) and oxalate (**k**–**o**) represent the primary components of CaOx stones and citrate (**a**–**e**) represents urine chemistry modulation via dietary countermeasures. 24-h urine volume is considered a common factor as the denominator in determining the relative concentration of the other three constituents. SI (**p**–**t**) is used to represent the integrated impact of these constituents. The color of each cell in the heat map represents the relative percentage of the population within that risk interval that exhibits the paired constituent values of the cell location on the heat map. Each heat map includes a nominal characteristic threshold for each constituent (dashed line) and the quadrant where both constituents contribute to a higher risk of renal stones in a terrestrial population (outlined by the solid line) per representative renal stone clinical risk levels as defined by the UT Southwestern Medical Center Stone Profile^92^. The characteristic threshold for SI is chosen based on published assessments of JESS CaOx SI calculations distinguishing SF and NSF populations derived from Rodgers et al.^93^. The color bar is scaled per urine constituent chemistry.

To investigate the relation of in-flight astronaut urine chemistry to predicted IRR, we examined the relative distribution of urine constituents of calcium, oxalate, urine volume, and citrate in each of the IRR risk categories illustrated as a family of constituent-paired heat maps shown in Fig. 5.

For IRR < 1 (leftmost column), astronaut excreted calcium and oxalate exhibit few instances where both calcium and oxalate exceed the clinical levels (3% of interval sub-population). As the magnitude of IRR increases, the proportion of the interval population that exhibits elevated calcium and oxalate increases from 20% in the 1 < IRR < 1.2 interval to the >65% in the >1.8 Interval, with the 49% point occurring in the 1.5 < IRR < 1.8 interval. Individually, a near majority of an interval population exhibits clinically elevated excretion for calcium (54%) or oxalate (49%) at and above the 1 < IRR < 1.2 intervals. Examination of the top 3 rows in Fig. 5 indicates that the in-flight astronaut 24-h urine volume is chronically low for a significant proportion of each risk interval population. Only the IRR ≤ 1 interval exhibits a significant proportion of the population (42%) with volume outputs above the clinical risk threshold of 2 L/day as compared to the next highest interval (1 < IRR ≤ 1.2; 19%). When considered in combination with calcium and oxalate at IRR > 1.2 intervals, significant proportions of the interval populations reside in clinically high-risk regions (lower right quadrant) of the heat map (volume and oxalate ≥ 56%; Vol and calcium ≥ 62%) and exhibit SI > 21. Citrate (top row), which NASA has considered as a potential in-flight countermeasure^30^, is shown with >92% of each interval population above the minimum clinical recommended level.

**Fig. 6:**
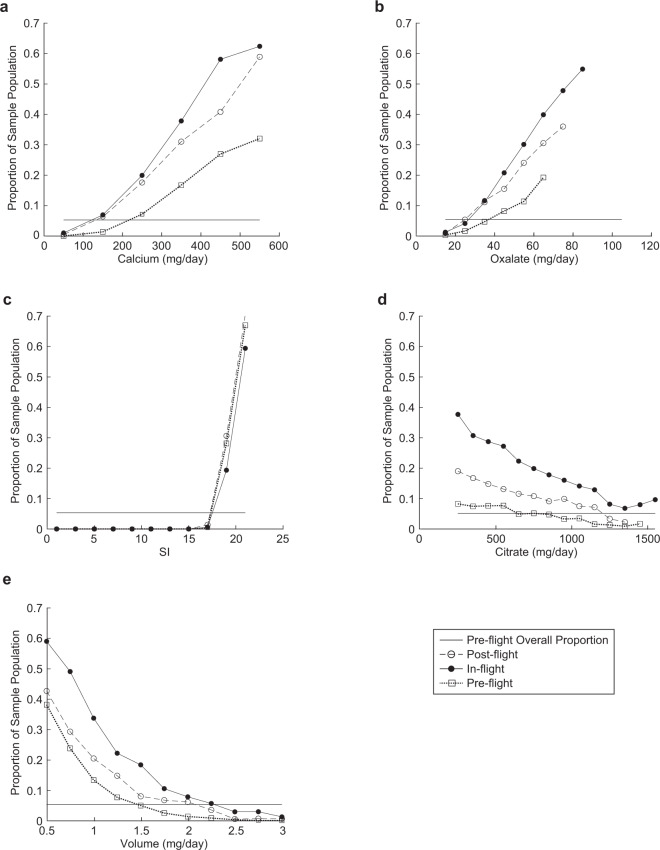
Plots of the proportion of the simulated astronaut population with IRR ≥ 1.2 with respect to 24-h urine levels. Each figure demonstrates the population proportion with IRR > 1.2 when evaluated independently for **a** calcium, **b** oxalate, **c** SI, **d** citrate, and **e** 24-h volume levels used in the in-flight, post-flight and pre-flight simulations. Points on each curve represent the midpoint of each bin range: **a** calcium ± 50 mg/day, **b** oxalate ± 10 mg/day, **c** SI ± 1, **d** citrate ± 100 mg/day, and **e** volume ± 0.125 L/day. These bin sizes ensure at least 100 simulated results reside in each datapoint to maintain a representation of the other stone formation factors. It is to be noted that the pre-flight IRR ≥ 1.2 population proportion level is illustrated by the solid horizontal line on each graph.

Predicted IRR ≤ 1.2 appears to be a natural cutoff level within this analysis for assessing the risk of CaOx stone formation in astronauts, as urine chemistries with IRR values in this range correspond to clinical and case/control risk characteristics of terrestrial non-stone-forming and pre-flight astronaut populations. Given this assumed threshold and our simulation results, an astronaut can therefore expect to exhibit an odds ratio of 4.66 in-flight and 2.48 post-flight for experiencing urine chemistries that would promote stone formation with respect to pre-flight. We use this natural cutoff to explore further the potential impact of interventions that mitigate negatively altered urine chemistry by evaluating the criteria needed to achieve the proportion of the astronaut population with IRR > 1.2 at or below 5.3% of the total population. Figure 6 illustrates predictions of the proportion of the astronaut population that would exhibit IRR > 1.2 across equal intervals of 4 urinary constituents (calcium, oxalate, volume, and citrate), as well as for the derived quantity SI. Assuming all other factors remain consistent within the representative astronaut distributions, the pre-flight astronaut population maintains the threshold at-risk population state with a volume output of 1.5 L/day within a resolution of the sampling bin width of ±0.125 L/day as described in Fig. 6. To meet the stated threshold of 95% proportion of the population with IRR < 1.2, the output volume level for in-flight and 1-year post-flight astronauts would need to maintain an output volume ≥2.25 L/day and 2.125 L/day, respectively. Pre-flight, excretion rates at or below the clinical risk boundaries of calcium = 250 mg/day, and oxalate = 45 mg/day meet the 95% population proportion threshold. Reducing the in-flight and post-flight calcium excretion rates by half of the pre-flight threshold level or the oxalate excretion rates to 28 mg/day results in population proportions that meet the 95% with IRR < 1.2 threshold. Pre-flight, population proportions exhibit insensitivity to citrate levels over 600 mg/day. In-flight citrate levels fail to independently reduce the proportion of the population to pre-flight threshold levels. However, a 10% population above the at-risk threshold can be achieved at citrate levels between 1200 and 1300 mg/day. Post-flight population proportions reduce to pre-flight target levels as citrate excretion approaches between 1100 and 1200 mg/day. The proportion of the population with IRR > 1.2 is near zero for urine chemistries with SI < 17, after which the proportion of the at-risk population increases significantly with increasing SI and in a nearly identical manner for each astronaut population. This interesting observation likely results from the trade-offs between thermodynamic (JESS) and physicochemical (PBE) effects resulting in smaller predicted free stream stone sizes until this supersaturation level is exceeded.

## Discussion

The occurrence of renal stones poses an in-flight astronaut health risk due to the impact of renal colic on human performance, mission supplies, mission timeline, and the added risk of an austere environment that could potentially lead to complications related to hematuria, infection, hydronephrosis, and sepsis^1^. Despite these hazards and studies that infer enhanced risk due to increased relative supersaturation of renal stone-forming salts^9,19–22^, a systematic means to weigh renal stone interventions and outcomes to other in-flight medical risks remain a significant challenge for the human spaceflight community. The model analysis workflow presented in this study utilizes computational simulations of CaOx free particle nucleation, growth, and agglomeration^47^ to characterize the risk of CaOx renal stone formation that flight-ready astronauts face relative to pre-flight expectations. By applying probabilistic numerical approaches to develop robust and quantitative analysis tools specially trained to address novel astronaut urine chemistries, we seek to provide spaceflight planning and decision-makers with a quantitative means to appraise astronaut renal stone risk mitigation alternatives intended to reduce CaOx stone formation risks in-flight and post-flight.

The analysis exhibits several limitations that should be considered when evaluating this study’s findings. Foremost of these is that we only consider the presentation of CaOx stones, which are estimated to be only 75% of terrestrial renal stones^13^ and 85% of renal stones presented by astronauts post-flight^9^. Therefore, when assessing the population renal stone risk to astronauts, we must assume that 15 to 25% of the total baseline incident renal stone risk may not be represented by this analysis even though the recommendations resulting from this analysis may extend, in part, to other types of stones. Similarly, we limit the training data to flight-ready astronauts prior to and 1-year post spaceflight and do not attempt to assess the potential variation in in-flight relative risk over the course of a mission, as well as report only averaged risk independent of mission duration. We do not consider recurrent stone formers whose recurrence rates may be orders of magnitude higher than healthy subjects^16,18^. We consider the astronaut population data as homogenous and preselected to be in the NSF clinical category^10,11^. Unless otherwise stated in the data processing, we neglect age, sex, race, and ethnic differences in the data. We also assume that astronauts, due to regular medical screening, are likely in better health and experience unique environmental factors not common to other populations at-risk for renal stones. There may be influences from these assumptions that potentially skew data locally or harbor further insights into mitigating specific crew risks that should be investigated in future studies.

By treating each urine constituent as an independently sampled factor, we did not retain potentially inherent correlations between individual urine components such as with calcium and pH^17,48,49^. To evaluate the impact of this assumption, we used the NHSI SF data set^45^ to assess the potential change in risk posture, assuming two important constituents were no longer sampled independently. Based on the significance of effects on CaOx stone formation reported in the literature^48,49^, a Spearman’s *ρ* correlation factor of 0.25, estimated from the correlation of pre-flight astronaut training data, was applied to the relationship between NHSI SF calcium (mg/day) and pH distributions. We then sampled the dependent distributions in conjunction with the remaining independently sampled urine constituents to reproduce the NHSI case population analysis. The effect of correlating the calcium and pH factor on the output produced a <1% change in IRR from the non-correlated case. Therefore, we assume that independent sampling adds minimal (<1%) uncertainty to our analysis. This may not be the case if this analysis is extended to other populations with different demographics or underlying urine chemistry distributions. Urine chemistry factors not included in our analysis, such as creatinine and other constituents associated with kidney function, may also modulate stone promoting effects with respect to risk assessment^45,50,51^.

The Poisson regression training process utilizes general population incidence rates and approximated astronaut sample population incidence rates, adequately characterizing the training populations. This assumption represents an inherent epistemic uncertainty in the analysis. Additionally, a greater number and spread of post-flight urine samples may bias the regression curve toward post-flight incidence rates. We potentially see this in the regression intercept incidence rate, which is comparable to that reported for all US Department of Defense >40 years of age populations rather than the aviator only averages that are assumed analogous to the astronaut base incidence rates^52^ because aviators are also selected to be NSF^53^. Additionally, limiting the maximum incidence rate prediction to avoid extrapolation outside the bounds of the training data potentially results in lower mean IRR predictions in the simulated populations.

In the application of the PBE model, we utilized a characteristic value, MSS, similar to that suggested by Kassemi and Thompson^36,37^ to capture important biochemistry and physical growth effects. However, a single factor associated with urine chemistry and nidus precipitation reaction may not adequately represent alternative stone formation processes contributing to clinical stone presentation, such as (1) hyperuricosuria contributing to the heterogeneous precipitation of CaOx crystals, (2) fixed-particle (Randell’s Plaque), and (3) anatomical (tubule, collecting ducts, and loop of Henle) features combined with flow-induced crystal-to-crystal interactions resulting in localized CaOx crystal deposition^18,47,54^. In the former case, hyperuricosuria, likely related to a high purine diet, potentially leads to urate crystals that act as substrates for CaOx deposition, changes in the local concentration of inhibitors and may alter pH balance to influence CaOx precipitant potentials^55–57^. In our analysis, the JESS ability to account for urate complexes (H^+^, Na^+^, K^+^, NH_4_^+^, and Ca_2_^+^) represents the primary means of including uric acid. Despite including urate complexes in the speciation analysis, JESS is reportedly insensitive to these changes over the range of astronaut uric acid concentrations^58^, implying that we have higher uncertainty in estimates of CaOx SI values where uric acid concentration is high. Elevated uric acid concentration may also influence estimates of MSS from the PBE model by altering the nucleation rate constant^36^. Estimates in the decline in the formation product ratio (FPR) of calcium oxalate with increased uric acid concentration^56^ infer a potential increase in the nucleation rate constant of ~38%, which would correspond to an ~10% increase in IR and IRR estimates for elevated concentrations of uric acid in the simulated astronaut population. In the latter case, computational studies using the PBE model coupled to computational fluid dynamics simulations indicate that variations in gravity level and orientation associated with spaceflight alter the CaOx crystal deposition and enhance the clearance of smaller crystals before significant growth can occur^54^. This complex interplay with respect to gravity is not captured by this current analysis and would lead to lower IRR predictions for in-flight astronauts than currently estimated. The use of single 24-h urine also precludes consideration of variations in urine concentration throughout the renal system, single void variations within the 24-h period, or day-to-day variations^59^. We contend that we implicitly include many of these aspects of the analysis through our statistical sampling of the real human data, as discussed above. The PBE simulation also produces MSS at discrete bin intervals representing a small uncharacterized uncertainty to the analysis at larger stone sizes.

We establish the overall performance of the analysis process utilizing several published population case and control studies, as shown in Figs. 2 and 3. Lacking a direct comparative astronaut referent, these comparative analyses act as a surrogate characterization of the analysis process by interrogating relative risk between terrestrial non-stone former (control) and stone former (case) populations and individual 24-h urine level constituents^45,60^. The inference is that we can expect a similar performance of our approach when examining the in-flight and post-flight astronauts (cases) relative to pre-flight astronauts (controls). When IRR is calculated with individualized control, the simulation analysis performs as expected, generating a unique IRR population distribution for both case and control urine chemistry data. In all instances, the case populations could be discriminated from the controls via observing IRR population statistics, as graphically depicted in Fig. 2. Specifically, controls exhibit lower mean IRR and much less skew than exhibited by case populations. Additionally, the maximum upper adjacent IRR of the controls only exceeded 1.20 by 3% in one instance, while all case populations exceed this value between 8% to 25%, illustrating the case population urine chemistries result in consistently elevated predicted risk levels.

To characterize the analysis process in evaluating the individual component impact on relative risk, we compare referent and predicted RR estimates evaluated over discrete urine chemistry ranges^45^. The RR indices utilized in these comparisons focus on those constituents with the most influence on our simulation outcomes and should not be considered to represent the entire spectrum of a urine constituent RR profile. Within the context of the referent binning ranges for the 24-h volume and daily excreted citrate, the trend and magnitude of the SF mean RR compares well to that of the referent, with the effect of increased volume producing a decrease in mean RR and citrate producing a relatively flat response with mean RR values generally below 1. This appears to be consistent with the published idealized performance of the PBE model to variations in citrate and 24-h volume associated with the specified ranges^37^. We note that both inhibitors predicted upper 95th CI in excess of the referent, which indicates the dependence of the RR estimates on the other urine chemistry constituents. Except for the 150–199 mg/day calcium range, calcium and oxalate produced expected trends of increasing RR with increasing excretion levels, although we found the predicted means and upper 95th CI lower than that of the referent. This difference may be attributable to the referent’s inclusion of factors not considered by our modeling analysis, such as other stone types besides CaOx, recurrent stone formers included in the case populations (NSHI 6%; HPFS 14%), and bin specific sample imbalances that may contribute to higher overall relative risk observations.

Comparing the analysis process predictions made using a selection of published non-astronaut population’s urine chemistries, we illustrate that the analysis can distinguish key population statistics between case (SF) and control (NSF) populations^45,60^. Further, the system predicts relative risk contributions of individual urine constituents of interest comparable to observed outcomes. These characterization findings support the application of this model analysis in distinguishing astronaut pre-flight to the in-flight and post-flight relative risk of CaOx stone formation.

Analysis of the representative astronaut urine chemistries identified key features of each flight-status population’s relative risk that were markedly like those found for NSF-SF referent populations in Fig. 2. The pre-flight control population, normalized to the predicted pre-flight incident rate mean in Fig. 4a, is sufficiently like referent non-stone-forming control populations to advocate its use as the risk analysis reference. The in-flight population produced the highest relative risk characterization with just over a fifth of the potential population exhibiting IRR greater than the high-risk demarcation limit of IRR > 1.2. The 1-year post-flight populations appear to nominally only return halfway toward the pre-flight baseline IRR, with over a 10th of the population remaining at IRR > 1.2. Unsurprisingly, clinically elevated calcium and oxalate excretion and low 24-h urine volumes, indicative of hypercalciuria as illustrated by elevated in Figs. 4d and 5 (bottom row), typify the majority of the proportion of the in-flight and post-flight population within the high-risk category. In both these elevated risk sub-populations, the citrate concentration remained generally elevated, inferring that the current variations in the astronaut community’s citrate levels produce a minimal change in relative risk posture. It should be noted that these observations would hold should another reasonable high-risk threshold IRR, such as IRR > 1, be chosen, with only the identified proportions differing.

Low-urine output is a common observation associated with increased astronaut risk of presenting most types of renal stones as reported in the spaceflight literature^1,9,15,19,20,28,50,59^, often followed with qualitative recommendations that increased fluid intake to achieve urine output levels >2.0 L/day to potentially mitigate stone formation. A challenge in spacecraft and mission design decisions lies in the ability to estimate how much the risk is reduced when such recommendations are totally or partially followed. Our findings support that chronic low-urine volume, associated with fluid shifts and limited liquid intake, results in elevated concentrations of Ca and Ox and exacerbate the astronauts’ in-flight and post-flight risk levels beyond that of the pre-flight population (Figs. 5 and 6). Further, we provided a quantitative approach to inform decisions about the management of astronaut CaOx stone risk using our analysis process to estimate at what levels of urine constituents would need to be modified to achieve the same proportion of the in-flight and post-flight astronauts with IRR < 1.2 as seen in pre-flight populations, i.e., to have the same odds of an astronaut having elevated renal stone risks before, during, and after a flight. In the case of 24-h urine volume, we determined prescribed levels of >2.25 L/day in-flight and >2.12 L/day post-flight resulted in an estimated mean SI value of <9.0 in both cases as indicated in Table 2, which summarizes the relative change in SI and overall risk for plausible operational prescriptions and our analysis recommendations. Assuming the insensible water losses on a spacecraft tend to the high end of nominal terrestrial values of 0.7–0.9 L/day^17^ due to lower spacecraft humidity levels^20,61^, we can estimate that in-flight astronauts should maintain a daily fluid intake from all dietary sources of >3.2 L/day and 1-year post-flight astronauts should strive to maintain a fluid intake of >2.9 L/day by extrapolating from the clinical recommendation of fluid intake to achieve protective levels of urine output^17,25^. This exceeds the current practice of 2.0 to 2.5 L/day fluid intake prescribed for in-flight astronauts^59,62^. It may be impractical to achieve both logistically and operationally, considering the resource limitations and daily schedules driven by the US spaceflight environment. Perhaps a more achievable goal is a nominal output of 1.75 L/day, with a corresponding intake of fluids between 2.5 and 2.7 L/day, as Fig. 6 shows this reduces the predicted proportion of high-risk astronauts to ~10% of the total population with a mean SI = 11.3.

**Table 2:** The mean and median SI, percentage of the simulated population with IRR < 1.2, and the change in that percentage from baseline for select plausible operational prescriptions and recommended mitigation approaches.

Plausible operational conditions or recommended mitigation	Nominal urine output per day	SI (mean)	SI (median)	Population with IRR < 1.2	Delta population with IRR < 1.2 without recommendation
Inflight					
(Baseline) prescribed in-flight water intake 2.0–2.5 L/day	1.10–1.8 L	13.62	11.97	81.92 %	NA
Water intake 1.7–1.9 L/day	1 L	18.96	16.63	65.59%	−16.33%
Water intake 1.95–2.15 L/day	1.25 L	15.05	13.43	77.88%	−4.04%
Water intake 2.2–2.4 L/day	1.5 L	13.34	11.67	81.70%	−0.22%
Water intake 2.45–2.65 L/day	1.75 L	11.28	10.15	89.53%	7.61%
Water intake 2.7–2.9 L/day	2 L	9.94	8.82	92.22%	10.30%
Water intake 2.95–3.15 L/day	2.25 L	8.84	7.80	94.41%	12.49%
Water Intake 3.2–3.4 L/day	2.5 L	7.77	6.87	97.14%	15.22%
Reduce mean Ca excretion to 190 mg/day	1.10–1.8 L	11.20	10.51	94.85 %	12.93%
Reduce mean Ca excretion to 150 mg/day	1.10–1.8 L	8.93	8.39	98.71%	16.79%
Reduce Ox excretion to 35 mg/day	1.10–1.8 L	11.04	10.61	94.23%	12.31%
Raise citrate excretion to 1050 mg/day	1.10–1.8 L	11.90	10.52	90.6%	8.68%
Water intake 2.5–2.7 L/day + reduce mean Ca excretion to 190 mg/day	1.8 L	8.91	8.59	99.12%	17.20%
Water Intake 2.5–2.7 L/day + reduce mean Ca excretion to 190 mg/day + reduce Ox output to 35 mg/day	1.8 L	7.43	7.50	99%+	18.08%
Post-flight					
Post-flight water intake 2.9 L/day	2.12 L	7.07	5.61	95.60%	13.68%

We premise these fluid intake recommendations on the assumption that calcium, oxalate, and citrate excretions remain at the levels described by the current data. A reduction in calcium or oxalate, or an increase in citrate would presumably alter the prescribed fluid requirements. Elevation in spaceflight calcium urine excretion is generally assumed to be due to increased resorption of bone in load-bearing skeletal regions^1,9,22^. Exercise in microgravity reduces the overall bone loss by promoting the remodeling of new bone and moderately mitigating resorption^19,20,32^. Bone health studies show that bone resorption markers and Ca excretion levels peak early in mission and drop-off as mission duration progresses past 110 days, with excretion approaching ~10% above pre-flight levels^32,63^. Given the limitation that our approach represents the average mission relative risk, irrespective of mission length, predicted in-flight Ca dependence is shown in Fig. 6a infers that the contribution of Ca excretion to CaOx stone risk results in ~40% population above threshold at <30 days and ~20% of population above threshold at >120 days^32^. Even as the Ca excretion approaches near pre-flight levels, the predicted proportion of the astronaut population exceeds the target threshold level by 15%. Although the predominant contributing component to elevated renal stone risk, other contributing risk factors such as reduced daily urine volume and elevated oxalate in the astronaut population data result in an in-flight excreted Ca level having a higher risk state than the same level pre-flight based on our analysis, i.e., Ca is a significant, but not an independent, risk parameter in establishing IRR in-flight. Our predictions point to the need of maintaining Ca excretion below 150 mg/day to achieve an average in-flight risk similar to pre-flight levels.

In 2013, the 2010 NASA Bone Summit Panel published a comprehensive set of recommendations to reduce the impact of spaceflight on astronaut skeletal health^64^. These recommendations, subsequentially supported by in-flight studies and analyses^19,20,31,65^, strongly emphasized the potential of bisphosphonates as a pharmaceutical countermeasure to diminish bone resorption and overall astronaut health risks. For CaOx renal stone risk, bisphosphonates likely normalize a low in-flight Ca excretion at all phases of the mission^1,31^. The 2010 Bone Summit panel also recommended that preference be given to long-acting intravenous bisphosphonate treatment due to obvious operational advantages. Long and short-acting bisphosphonates have proven efficacy to reduce calcium excretion of greater than 2 years^66,67^, suggesting that in the case of long-acting bisphosphonates, subsequent in-flight dosing may be avoided for missions <3 years. Long duration bed-rest studies (>90 days) using long-acting intravenous bisphosphonates demonstrate Ca excretion levels below 150 mg/day are possible for significant periods of unloading^68^, which our analysis suggests would return the in-flight risk to pre-flight levels. The most recent report of an in-flight study with short-term bisphosphonates intervention combined with exercise demonstrated Ca excretion diminished to 210 ± 85 (SE) mg/day (>120 days)^32^. Short-term terrestrial control studies infer that a reduction in urine calcium excretion of 45–49 mg/day^69,70^ is likely with any bisphosphonate treatment and appears to be consistent with in-flight observations to within the observed standard error^32^. Taking the 45–49 mg/day reduction in in-flight Ca excretion as the minimum average benefit achievable by a bisphosphonate intervention, our estimates indicate that this reduces the predicted proportion of high-risk astronauts to <15% of the total population. When the 45–49 mg/day reduction is combined with a recommended 2.5–2.7 L/day fluid intake, we predict that >98% of the in-flight population will exhibit IRR < 1.2 (Table 2).

A potential option for controlling the CaOx stone risk is to reduce the concentration of excreted urinary Ox^71^. Ox excretion is a tightly controlled phenomenon in the kidney with tubule absorption working to keep serum Ox levels constant^72^. Approximately 65% of oxalate urine excretion is driven by dietary factors, including the amount of dietary calcium, which binds with oxalate in the gut before absorption^71,73^, forming insoluble crystalline CaOx that is eliminated in the fecal stream. However, the dietary absorption of Ox is variable between individuals on similar diets^74^. Individuals with elevated potential for Ox absorption can see as much as a 50% elevation in urinary excreted oxalate with a dietary calcium-to-oxalate ratio change from 4 to 1.6^75^. Dietary considerations must be balanced with other in-flight health risks^76,77^ and an oxalate-controlled diet may be clinically unwarranted without a diagnosis of secondary hyperoxaluria^78^. Should interventions be pursued, our analysis would suggest targeting a reduction in excreted oxalate to nominally 35 mg/day, which is ~10 mg/day higher than what recent research indicates for increasing terrestrial risk^75^. This recommendation reduces the predicted proportion of high-risk astronauts by more than half, such that 94% of the population exhibits an IRR < 1.2 (Table 2). In combination with 2.5–2.7 L/day increased volume recommendation, our simulations suggest reducing oxalate would result in 98% of the population with IRR < 1.2 (mean SI = 8.7). Including reduced excreted urine calcium recommendations in the simulation results in >99% of the simulated population with IRR < 1.2 (Table 2).

In determining the recommended interventions to produce in-flight risk levels equivalent to pre-flight risk thresholds, as summarized in Table 2, we consistently find that the astronaut population mean and the median CaOx SI must be at or below 9.0, and per findings illustrated in Fig. 6, population maximum should not exceed 17. This is consistent with the mean SI levels seen in the terrestrial population control characterization simulations shown in Fig. 2b and supports the importance of relating both thermodynamic and physicochemical effects to provide insight into risk reduction strategies. In terms of risk reduction countermeasures, both analog^79^ and flight^30^ studies have established the potential CaOx stone risk reduction benefits of potassium citrate when applied as a prophylactic countermeasure to raise urine citrate levels and reduce CaOx supersaturation in astronauts. As an inhibitor of stone risk, citrate increases urine pH, decreases Ca ion activity, CaOx supersaturation, and influences the local urine environment around the surface of the CaOx crystal, changing aspects of the crystal nucleation, growth, and aggregation^42,80–82^. This has led to its consideration as an in-flight countermeasure^1,30^. Our findings suggest that citrate excretion levels now achieved for in-flight and post-flight astronauts exhibit near its maximum available benefits. Our analysis shows that CaOx risk cannot be eliminated by increasing citrate within the range exhibited by the astronaut representative urine chemistry distributions. Reducing the predicted at-risk population by half with mean SI = 11.6 may be achievable at excretion levels around 1350 mg/day, ~4 times the clinical risk level, and a >60% increase in the current nominal levels. Examination of parametric evaluations with the PBE model, which accounts for these factors, illustrates that if citrate levels were allowed to drop below levels currently exhibited by the preponderance of astronaut urine chemistries, a nonlinear increase in the predicted MSS and subsequent IR and IRR would result^37^. With this observation, our findings suggest that combining increased citrate above current nominal levels with our other recommendations results in insignificant changes in the proportions of the at-risk in-flight population. Therefore, the use of potassium citrate is warranted as an in-flight countermeasure only to maintain current excreted citrate levels so as not to contribute to increased renal stone risk with respect to our other recommendations.

In this study, we characterized the increased CaOx renal stone incidence rates for astronauts and quantified the enhanced in-flight and post-flight relative risk compared to pre-flight levels. Our computational model is an integrated framework combining a PBE model involving thermochemistry, kinetics, and fluid physics with a probabilistic analysis utilizing 1517 astronaut 24-h urine chemistries. We identified that IRR = 1.2 calculated with our approach is a rational threshold risk of astronaut CaOx stone formation, as derived from our finding that urine chemistries with IRR < 1.2 correspond to clinical and case/control risk characteristics of terrestrial NSF and pre-flight astronaut populations. Our model enables us to make several notable observations and recommendations important to the space medical community, including quantitatively assessing that in-flight risk can be reduced by 50% through increasing water intake by 0.5 L/day or by 25% through decreasing calcium excretion by 45 mg/day via the reduction of bone resorption. Our simulations predict that in-flight fluid intake alone would need to increase from current prescriptions of 2.0–2.5 L/day to ~3.2 L/day to approach the CaOx IRR of the pre-flight population. Similarly, bone protective interventions would reduce CaOx risk to pre-flight levels if average Ca excretion alone is reduced from 240 to <150 mg/day, or alternatively, if the current in-flight average Ca excretion levels are diminished to 190 mg/day in combination with increasing fluid intake to 2.5–2.7 L/day. Further, the model successfully characterized the impact of current potassium citrate countermeasures in modulating the renal stone risk. Nevertheless, no amount of excreted citrate was predicted to be sufficient to return in-flight astronauts to pre-flight risk levels. As one of the few quantitative approaches to assessing in-flight and post-flight CaOx renal stone formation risk in astronauts, this analysis has the potential to provide a substantive influence on vehicle and mission designers in striking a critical balance between engineering and astronaut health requirements.

## Methods

### Prediction model design

Our study was reviewed by the NASA IRB at Johnson Space Center and received a determination of “Not Human Subject Research” (NASA IRB Study No.: STUDY00000437), indicating that model analysis and retrospective data used did not require NASA IRB approval as the effort did not involve the collection of data, did not use or produce identifiable or private information in the analysis, did not use astronauts as a test article and the acquisition of the retrospective data available from the NASA Lifetime Survey of Astronaut Health (LSAH)^83^ followed all applicable ethical, legal, NASA, and informed consent requirements. The LSAH also reviewed the final products of this analysis to verify the analysis results remained unidentifiable to insure astronaut privacy.

**Fig. 7:**
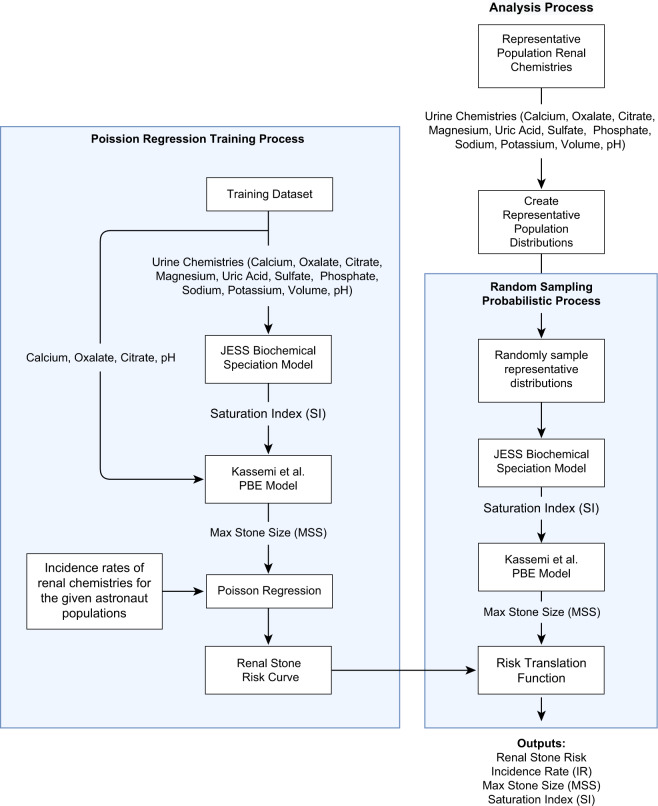
Illustration of renal stone incidence rate prediction model training and analysis processes. The left-hand side of the figure illustrates the use of individualized urine chemistries in sequential calculations of SI and PBE-MSS, known stone-forming characteristics (stone former, non-stone former), spaceflight status characteristics (pre- and post-flight), and estimates of the appropriate population incidence distributions (Fig. 1) in order to develop an MSS to IR relation utilizing Poisson regression. The right-hand side of the figure illustrates a similar process for the analysis, where representations of urine constituent population statistics are used to generate >10,000 unique urine combinations from which SI, MSS, and IR calculations are combined in a Monte Carlo process to predict the astronaut population risk.

Figure 7 illustrates the components and operational processes of the astronaut renal stone incidence rate prediction model that is used for training and analysis of CaOx incidence rate (IR). The model is implemented in MATLAB. For training, as illustrated on the left-hand side of Fig. 7, the model requires individualized urinalysis data attributed to populations with estimated initial stone-forming rates. In the analysis process, as illustrated on the right-hand side of Fig. 7, a population of interest is characterized by statistical representations of the urine constituents, which allows the generation of many thousands of potential combinations of unique urine chemistries in a Monte Carlo sampling process. Both training and analysis processes supply individual (actual or numerically sampled) urine chemistries to the chemical speciation tool (JESS^39^) for estimating the CaOx supersaturation. This is then provided as input to the PBE model^36,37^ to obtain characteristic stone size parameters. In the training process, we correlate the characteristic stone size parameters to the predicted IR of renal stones via a Poisson regression model. In the analysis, we process Monte Carlo sampled urine chemistries to predict MSS, then translate MSS to IR to characterize the sample population CaOx renal stone risk for the representative astronaut population. The following sections describe the data, primary model components, model training routines, and model analysis testing details.

### Data source

The data query to the LSAH requesting urine chemistries obtained from pre-flight flight-ready, in-flight, and post-flight astronauts resulted in a data set of 1517 urine samples from 581 individual astronauts. The pre- and post-flight samples included both shuttle and ISS astronaut urine samples, while the in-flight samples included solely ISS data. The information also included the day the sample was taken relative to flight, the number of days between an individual’s successive urine samples, and details regarding the number of days the sample was taken with respect to a pre-flight or post-flight stone incidence. Of the 1517 urine samples, 508 pre-flight and 433 post-flight (total: 941) included all the chemical component concentrations and measurements required to train the simulation-based analysis process: calcium, oxalate, citrate, magnesium, uric acid, sulfate, phosphate, sodium, potassium, volume, and pH. Table 1 details the population statistics for this pre-flight and post-flight model training data sets, respectively. Tabulated post-flight urine samples were collected solely from astronauts within one year of return from spaceflight. SF urine chemistries with stone occurrences within 5 years prior to the spaceflight were excluded. Similarly, urine samples after a post-flight stone occurrence were excluded. In-flight samples were excluded from the training data set.

The remaining 560 urine samples lacked data on at least one urine constituent required to perform individual analysis. Rather than discard this data, data for each constituent was independently combined and used as the basis for representing astronaut urine population statistics for the Monte Carlo analysis. The pre-flight test data set included 257 samples, 119 in-flight samples taken during ISS missions, and 184 post-flight samples. Table 3 illustrates the normal statistics for this characteristic astronaut analysis population data set used to represent pre-, in-, and post-flight populations.

**Table 3:** The mean, number of samples per measurement, and standard deviation of urine measurements used for testing the model.

	Pre-flight urine			In-flight urine			Post-flight urine		
	Mean (mg/day)	Standard deviation (mg/day)	No. of samples	Mean (mg/day)	Standard deviation (mg/day)	No. of samples	Mean (mg/day)	Standard deviation (mg/day)	No. of samples
Concentrations									
Calcium	190.0	107.9	243	241.5	107.6	120	190.6	118.9	148
Oxalate	36.3	11.5	95	44.7	18.5	116	36.6	16.1	28
Citrate	753.9	290.6	98	784.7	338.5	116	682.2	279.2	33
Magnesium	104.8	37.4	243	118.9	40.4	120	90.6	38.2	148
Uric acid	643.7	204.8	161	556.0	296.1	51	587.1	216.4	149
Sulfate	2207.8	844.4	94	2078.2	799.7	116	2129.2	1447.7	18
Phosphate	1042.4	354.6	229	1170.4	357.7	120	913.8	344.4	136
Sodium	4771.6	3466.7	240	3636.5	1154.6	120	4398.5	5554.3	142
Potassium	3770.4	2666.2	242	2647.6	923.4	120	3273.2	2741.1	149
Additional urine characteristics									
Volume (L)	2.1	1.0	257	1.6	0.8	120	1.8	1.1	175
pH	6.0	0.4	94	6.1	1.2	116	6.0	0.5	20
Total no. of samples			257			120			176

### Speciation of urine chemistry

The speciation code, JESS^39,43,84^ is used to calculate the chemical equilibrium distribution of component concentrations within the urine with a user-specified “no-precipitates” imposed constraint. Speciation, for the training and analysis activities, utilizes the individualized actual or sampled astronaut urine chemistries and characteristics, respectively, to establish the free ion concentrations and the CaOx JESS SI^80,84^. As noted in Rodgers et al.^43^, SI is an equivalent type of measure of relative supersaturation (RSS) as it is calculated according to the same physicochemical principles as that used in EQUIL2, with the additional consideration of phosphate species interactions and superior characterization of citrate speciation^44^.

### Characteristic stone growth in a free stream

We used a MATLAB 2010© implementation of the PBE model, developed by Kassemi and Thompson^36,37^, to characterize the stone growth potential of each of the training and sampled analysis urine chemistries. As an analogy to the stone formation in the kidney, the PBE model tracks the formation and growth of CaOx stones using the mathematical framework of a mixed suspensions mixed product removal crystallizer that is represented by an integro-differential equation in terms of the crystal diameter-based population density distribution. The formulation and methodology assume that the growth rate is independent of crystal diameter, that agglomeration of crystals conserves particle diameter rather than volume, and the nucleation and growth deplete the local ionic concentrations following a simple mass balance in a free stream of urine^36,37^. Utilizing the initial conditions of SI, pH, and ion concentrations of calcium, oxalate, and citrate obtained from the chemical speciation calculations, the PBE model iteratively solves a closed set of equations for nucleation, growth, agglomeration, and mass conservation to predict the steady-state diameter distribution of CaOx crystals. The distribution of predicted stone particle diameters effectively characterizes the free stream potential for precipitation and the evolution of CaOx stones for specific biochemistry. Given that operationally, the risk of an adverse formation of a renal stone will likely correspond to larger stone diameters, we further characterize the PBE model results using the largest single stone diameter predicted in 1 mL of urine. We refer to this characteristic value as the maximum stone size (MSS).

In both the training and analysis paths, we utilized the same parameters for nucleation rate, linear growth rate, agglomeration kernel, and species solubility, as reported by Kassemi and Thompson^36,37^. We rely on the model verification and validation performed by Kassemi and Thompson^36,37^ as confirmation that the PBE model has been tested for adequate fidelity within the context in which we apply it in this study. With respect to PBE model’s sensitivity, 0.07% of the simulations using the astronaut population analysis data failed to converge when concentrations approach values that are not physiologically representative. Such combinations occur when the urine chemistry sampling simultaneously captures the extremes of the distributions for multiple parameters. We have excluded these trials from the probabilistic simulation as indeterminate results.

### Estimating symptomatic calcium-based kidney stone incidence and recurrence rates

A study by Porter and Rice^52^ identifies military aviators as experiencing an average stone incidence rate of 4.40 per 1000 person-years, which is similar to the incidence rate for a Houston, TX-based NASA astronaut analog population of 4.2 per 1000 person-years^85^. Assuming, per Kittanamongkolchai et al.^13^, that the primary constituent of ~86% of symptomatic stones is calcium and 87.1% of those are CaOx stones, the Porter and Rice^52^ incidence rates are slightly below that of the incidence rate utilized in the NASA Integrated Medical Model^1,86^. Given analogous activities and stressors between aviators and astronauts evident by the similarities in predicted initial occurrence rates, we utilize the Porter and Rice^52^ incidence information augmented by proportions of reported primary calcium stones derived from Kittanamongkolchai et al.^13^ (87.1% of CaOx stones to measured stones), represented as a Gamma Probability Density Function (PDF), shown in Table 4, as a well pedigreed and reasonable means of estimating the (5-year) pre-flight astronaut incidence rate for primary calcium type stones.

**Table 4:** Estimated incidence and recurrence rates of calcium-based symptomatic kidney stones (rates in events per 1000 person-years).

	Statistics		95th CI		
Domain - Ca stones	Mean	std	2.5th Percentile	97.5th percentile	Estimated proportion of CaOx stone type
Overall men and women^13^	2.54E+00		2.42E+00	2.67E+00	8.71E−01^13^
All Astronauts as of 2004 All stone types Pre/in/post-flight^9^	2.67E+00	7.01E−01	1.48E+00	4.21E+00	7.49E−01^9^
NASA IMM estimated rate^1^	3.65E+00	3.75E−01	2.92E+00	4.39E+00	1.00E+00^1^
Pre-flight astronaut non-stoneformer Aviator assumption^52^	3.79E+00	8.65E−02	3.62E+00	3.96E+00	8.71E−01^13^
Astronaut 1-year post-flight incidence (see “Methods” section)	1.73E+01	6.33E + 00	8.33E+00	2.88E+01	8.71E−01^13^
Recurrent stone former 1 prior stone^16^	5.61E+01	6.41E + 01	5.43E−01	2.32E+02	8.71E−01^13^
Recurrent stone former ≥2 prior stones^16^	1.76E+02	2.68E + 02	5.70E−02	9.47E+02	8.71E−01^13^

To assess a reasonable representation of renal stone occurrence rate post-flight, we utilize a Bayesian updated process with an informed prior reasoned from published studies of pre- to post-flight urine chemistry changes updated with observed post-flight occurrences of symptomatic stones. We followed a process similar to that described by Christensen et al.^87^ for determining post-flight gamma prior parameters a and b (Table 4) from an estimate of the most likely value (mode) and 95th percentile derived from currently available information. Focusing on the risk associated with changes in calcium type supersaturation, a review of Whitson et al.^15^ and Pietrzyk et al.^9^ suggests that astronauts exhibit an increase of between 1.36 and 1.8 in pre- to post-flight renal stone risk, respectively, (avg. 1.58). We use this average value with the estimated average pre-flight incidence rate for stones whose primary constituent is calcium, given in Table 4, to determine a representative most likely incidence rate. We assigned this value as the mode of a representative Gamma distribution prior. Kassemi et al.^36,37^ state that based on the results of the PBE based model, idealized in-flight and immediate post-flight astronaut urine chemistries are predicted to perform similarly to Earth-based stone formers. Extending this analogy to incidence rate, we assume that the upper limit of the incidence post spaceflight should not exceed a rate representative of recurrence in 1-g stone formers. A recent study by Ferraro et al.^88^ consolidates input from 21 randomized control trials investigating the recurrent calcium-based renal stone occurrence. It indicates that the median rate of calcium constituent stones, both asymptomatic and symptomatic, falls at 60 events per 1000 person-years for persons having only one previous stone event. Based on Kittanamongkolchai et al.^13^, we assume that 14% of all symptomatic and asymptomatic stones are asymptomatic. We assigned the Gamma prior 95th percentile to be the combination of the median rate from Ferraro et al.^16^, adjusted with the aforementioned estimated proportion of asymptomatic and symptomatic stones.

Sibonga and Pietrzyk^1^ state that 7 symptomatic renal stones have occurred in astronauts within one-year post-flight (i.e., in 358 person-years). Since the variation in the composition of the renal stones experienced by astronauts remains unclear per Pietrzyk et al.^9^, we assume that proportions of calcium stones in all astronaut symptomatic stones are used in our NSF incidence rate assessment continue to apply^13^. This implies that only 6 of the 7 one-year post-flight astronaut stones exhibit a primary calcium constituency. Table 4 illustrates the estimated post-flight incidence rate as the posterior of the Bayesian update analysis utilizing observed 1-year post-flight incidence to update the informed conjugate Gamma prior under the assumption that the occurrence follows a Poisson process.

### Poisson regression

The training data was used in a Poisson regression for rates methodology^87^ to develop a continuous relation of PBE-MSS to renal stone incidence rates that can be used in the risk analysis calculation. The MSS from the renal chemistry in the training data set is correlated with the known distribution of the subject’s renal stone incidence rate distribution, based on their stone-forming status and population characteristics. Table 4 lists the discrete stone-forming status populations available for this analysis and the estimated mean and uncertainty of each corresponding population incidence rate.

The training data set of pre- and post-flight astronaut urine samples contain individuals that can be considered non-stone former (NSF) and stone former (SF) astronauts. Suppose individual astronaut chemistry had no known history of renal stones 5 years prior to flight. In that case, the pre-flight, NSF incidence rate distribution derived in Table 4 is used for that chemistry. The post-flight urine data is limited to those urine chemistries obtained within one-year post-flight.

Because this model focuses on the first occurrence of renal stones, and not on recurrent stone formers, we excluded 16 astronaut samples obtained within 5-years post-presentation of a symptomatic renal stone. This assumes that despite ongoing interventions, samples obtained from these participants would be representative of high variance re-occurrence rates^16^. In addition, we excluded samples after stone formation within and beyond 1-year post-flight based on similar assumptions regarding uncertainty and likely single stone recurrence rates.

**Fig. 8:**
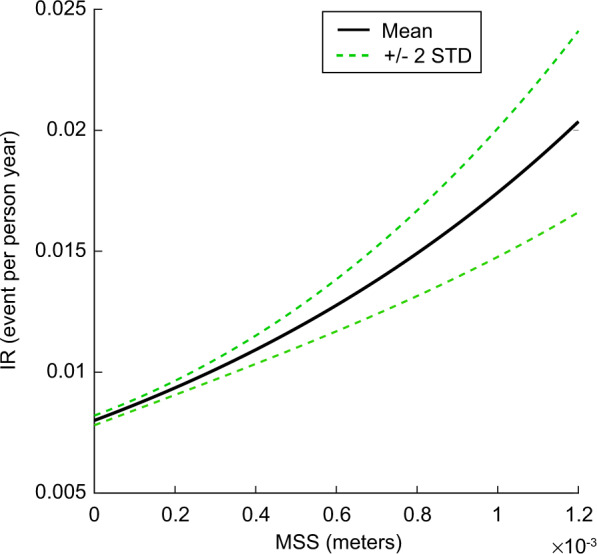
Relationship of IR to MSS as determined via Poisson regression for rates. The largest MSS for a complete pre- and post-flight data set is 1.2 × 10^−3^ m. Rather than extrapolating, we assign the max calculated incidence rate of the regression when the MSS exceeds the limits of the training data. Therefore, to keep the model within the fit’s limits, the incidence rate output is not reported as greater than at 2.07 × 10^−^^2^ person-years. All IRR are calculated by dividing the discrete, predicted, IR values by the appropriate reference population predicted IR mean value. The parameter values for the resultant curve of the regression are *A* 8.0027 × 10^−3^ person-years, and *B* 7.7804 ×10^2^ (1/m).

Figure 8 shows the resultant Poisson regression function relating the PBE-MSS with population incidence rates. Our implementation of the Poisson regression for rates^87^ uses the following process: Using a fixed time interval (100,000 person-years), we utilize the incidence rate distribution shown in Table 4 to estimate the number of incidences for each corresponding PBE-MSS calculated from the individual training data urine chemistry. We then fit a curve to this data via Poisson regression. The process is repeated up to 10,000 times, each time randomly sampling for a unique rate for the incidence rate distributions. We aggregate the resultant family of curves and perform relevant statistics to represent the aggregate function by the exponential equation

1 $${{{\mathrm{IR}}}}\left( {{{{\mathrm{incidence}}}}\;{{{\mathrm{per}}}}\;{{{\mathrm{person}}}} - {{{\mathrm{year}}}}} \right) = A \ast e^{B \ast {{{\mathrm{MSS}}}}}$$

where *A* and *B* are coefficients of the regression. The resultant Poisson regression curve (Fig. 8) is used in the astronaut renal stone risk analysis.

### Assessing renal stone risk: validation and analysis

Before addressing astronaut risk, we evaluated and characterized the analysis process path illustrated on the right-hand side of Fig. 7 through a comparative analysis using published urine constituent distributions of SF (case), and NSF (control) paired population studies. Recall that to perform an analysis, the input distributions for each urine constituent are treated as an independent parameter, represented by a gamma distribution matching the reported statistics. Within the analysis, a Monte Carlo step creates 10,000 or more random unique urine chemistry combinations that are then processed through JESS, the PBE model, and then the Poisson regression curve in Fig. 8, to calculate the SI, MSS, and the corresponding estimate of CaOx IR per person-year.

### Validation referent data sets and methods

We utilized the SF (case) and NSF (control) 24-h urine data from Parks and Coe^60^ and Curhan^45^ as referent population data sets to assess model performance, indicated as [A] and [B], respectively, in Fig. 2. The data published by Parks and Coe^60^ includes male and female participants in the age range of 20–55. From the Cuhran^45^, we utilized female NHSI population data and male HPFS population data. These data exhibited an average age of 61 and 59, respectively, and included the contribution of the relative risk of kidney stones from urine constituents important in the formation of calcium-based stones. We note that Parks and Coe^60^ data lacked sulfate information. Yet, to still utilize this data set, we applied the corresponding NSF or SF sulfate values from NHSII and HPSI to complete the female and male validation data sets, respectively. For the purpose of model characterization, we fit each urine constituent in these studies to a representative gamma distribution that was sampled and used in the analysis.

### Astronaut risk analysis

The analysis estimating astronaut CaOx renal stone risk followed the approach used in the comparative validation step, using pre-flight, in-flight, and post-flight test data sets (Table 3) as individual input to assess the change in relative risk of each phase of an astronaut’s flight available status. The majority of the astronaut core is slightly younger than the validation cases with the average age of candidates being 34 with a range of 26–46^89^ and age astronauts at last flight being 45.29 with a max. of 61, discounting John Glenn’s record-setting flight in 1998 at the age of 77^90^. We further assume that due to enhanced medical surveillance, the astronauts’ health is likely well characterized throughout their careers^89^. As our post-flight data is only taken within a year of return from space, we reasonably assume the data is representative of an average 40–50 years-old population. As of June 2013, only 57 of the 534 people who had flown in space were female, so we can infer that our aggregated data is skewed toward males^91^.

### Statistical techniques

We describe the majority of the statistical techniques used in the modeling system, such as data distribution estimates, performing Poisson regression, and the Monte Carlo sampling, as part of the various methods sections where they are employed. Post-processing analysis identifies statistical characteristics of sub-populations of the predicted population, such as mean, SD, and skew, using standard techniques. Statistical comparison for the characterization and validation tests uses a two-tailed *z*-test as outlined by Altman and Bland^46^ for the comparison of relative risks with large *n*. Comparison test between pre-, in-, and post-flight astronaut risk distributions utilize two-tailed *z*-test, as n of each distribution is large.

### Reporting summary

Further information on research design is available in the Nature Research Reporting Summary linked to this article.

## Supplementary information

Reporting Summary

## Data Availability

Individualized astronaut urine chemistry data are considered protected due to the privacy act. The de-identified, individualized astronaut data used in this study can be requested from the NASA Lifetime Survey of Astronaut Health, part of the NASA Life Science Data Archive, at https://lsda.jsc.nasa.gov/Home/Index. Please refer to request ID #: 10658 for the specific data set used in this study.

## Code availability

The PBE model code described in the methods is from previously published sources^36,37^. All code developed to perform statistical assessments and Monte Carlo simulations can be obtained with a reasonable request to the NASA affiliated authors and after appropriate government export control review.
