# Supplementary material for: Numerical characterization of astronaut CaOx renal stone incidence rates to quantify in-flight and post-flight relative risk
Source: NPJ Microgravity. 2022 Jan 28;8:2. doi: 10.1038/s41526-021-00187-z (PMC8799707; doi:10.1038/s41526-021-00187-z)
Supplement: Supplementary file 1 — Reporting Summary [file 41526_2021_187_MOESM1_ESM.pdf]

nature research

Corresponding author(s): Jerry G. Myers, Jr.

Last updated by author(s): Nov 7, 2021

Reporting Summary

Nature Research wishes to improve the reproducibility of the work that we publish. This form provides structure for consistency and transparency in reporting. For further information on Nature Research policies, see our Editorial Policies and the Editorial Policy Checklist.

Statistics

For all statistical analyses, confirm that the following items are present in the figure legend, table legend, main text, or Methods section.

n/a	Confirmed	
☐	☒	The exact sample size (*n*) for each experimental group/condition, given as a discrete number and unit of measurement
☐	☒	A statement on whether measurements were taken from distinct samples or whether the same sample was measured repeatedly
☐	☒	The statistical test(s) used AND whether they are one- or two-sided<br>*Only common tests should be described solely by name; describe more complex techniques in the Methods section.*
☐	☒	A description of all covariates tested
☐	☒	A description of any assumptions or corrections, such as tests of normality and adjustment for multiple comparisons
☐	☒	A full description of the statistical parameters including central tendency (e.g. means) or other basic estimates (e.g. regression coefficient) AND variation (e.g. standard deviation) or associated estimates of uncertainty (e.g. confidence intervals)
☐	☒	For null hypothesis testing, the test statistic (e.g. *F*, *t*, *r*) with confidence intervals, effect sizes, degrees of freedom and *P* value noted<br>*Give P values as exact values whenever suitable.*
☐	☒	For Bayesian analysis, information on the choice of priors and Markov chain Monte Carlo settings
☒	☐	For hierarchical and complex designs, identification of the appropriate level for tests and full reporting of outcomes
☒	☐	Estimates of effect sizes (e.g. Cohen's *d*, Pearson's *r*), indicating how they were calculated

Our web collection on statistics for biologists contains articles on many of the points above.

Software and code

Policy information about availability of computer code

Data collection	No data collection software was used by these authors
Data analysis	Data analysis was performed primarily using MATLAB, JESS and MS-Excel. MATLAB built in features used to produce Monte Carol sampling and perform Poisson Regression. A MATLAB wrapper code was developed to automate JESS usage. PBE model in MATLAB provided by developer.

For manuscripts utilizing custom algorithms or software that are central to the research but not yet described in published literature, software must be made available to editors and reviewers. We strongly encourage code deposition in a community repository (e.g. GitHub). See the Nature Research guidelines for submitting code & software for further information.

Data

Policy information about availability of data

All manuscripts must include a data availability statement. This statement should provide the following information, where applicable:

- Accession codes, unique identifiers, or web links for publicly available datasets
- A list of figures that have associated raw data
- A description of any restrictions on data availability

Individualized astronaut urine chemistry data is considered protected due to the privacy act. The de-identified, individualized astronaut data used in this study can be requested from the NASA Lifetime Survey of Astronaut Health, part of the NASA Life Science Data Archive, at https://lsda.jsc.nasa.gov/Home/Index. Please refer to request ID #: 10658 for the specific data set used in this study.

Field-specific reporting

Please select the one below that is the best fit for your research. If you are not sure, read the appropriate sections before making your selection.

☒ Life sciences ☐ Behavioural & social sciences ☐ Ecological, evolutionary & environmental sciences

For a reference copy of the document with all sections, see nature.com/documents/nr-reporting-summary-flat.pdf

Life sciences study design

All studies must disclose on these points even when the disclosure is negative.

Sample size	there were 1571 unique urine samples obtained from 581 individual astronauts in different flight states. To perform the analysis sub-populations of this data were used for training (941) and simulation analysis (560). Not all urine constituents were provided in each simulation analysis sample thus table 3 gives the break down of sample count on a constituent and flight state basis.
Data exclusions	Any data received outside the 5-year pre-flight and 1 year post flight was not included in the study and not counted in the sample size count.
Replication	No specific measures were taken by this team to replicate data. De-identified data is from a astronaut surveillance and individual research studies as captured in the NASA Life Science data archive.
Randomization	N/A
Blinding	the data was de-identified, so we do not know the individual astronaut or the exact duration of each astronauts flight or the mission.

Reporting for specific materials, systems and methods

We require information from authors about some types of materials, experimental systems and methods used in many studies. Here, indicate whether each material, system or method listed is relevant to your study. If you are not sure if a list item applies to your research, read the appropriate section before selecting a response.

Materials & experimental systems

n/a	Involved in the study	
☒	☐	Antibodies
☒	☐	Eukaryotic cell lines
☒	☐	Palaeontology and archaeology
☒	☐	Animals and other organisms
☐	☒	Human research participants
☒	☐	Clinical data
☒	☐	Dual use research of concern

Methods

n/a	Involved in the study	
☒	☐	ChIP-seq
☒	☐	Flow cytometry
☒	☐	MRI-based neuroimaging

Human research participants

Policy information about studies involving human research participants

Population characteristics	Active Astronauts, i.e. individuals chosen to meet NASA standards for astronaut duties including meeting NASA health standards. The individuals are likely to have elevated surveillance of health issues as well.
Recruitment	As the data is from surveillance and approved research studies, the NASA LSA and LSDA acquires approval statements as needed based on previous approvals or lack thereof from individual astronauts.
Ethics oversight	NASA IRB determination of “Not Human Subject Research” Study No.: STUDY00000437; FWA No.: 00019876, Nov 5, 2021

Note that full information on the approval of the study protocol must also be provided in the manuscript.
